# An Underwater Polarization Image Fusion Algorithm Based on Information Entropy and a Hierarchical-Adaptive Fusion Framework

**DOI:** 10.3390/s26103231

**Published:** 2026-05-20

**Authors:** Fuqiang Wang, Wei He, Shanwei Ye, Ang Ma, Xichuan Zhou, Zonghuan Guo, Jianchao Wang, Lin Zhou, Yingcheng Lin

**Affiliations:** 1 School of Microelectronics and Communication Engineering, Chongqing University, Chongqing 400044, China; 202412131172@stu.cqu.edu.cn (F.W.); hewei007@cqu.edu.cn (W.H.); ysw@stu.cqu.edu.cn (S.Y.); mang349430@outlook.com (A.M.); zxc@cqu.edu.cn (X.Z.); 2 Seres Group Co., Ltd., Chongqing 400038, China; zonghuan.guo@seres.cn (Z.G.); jianchao.wang257080@seres.cn (J.W.)

**Keywords:** image fusion, Gaussian decomposition, underwater image enhancement, information entropy

## Abstract

Underwater images often exhibit low contrast and loss of detail due to light scattering and absorption, which poses significant challenges for visual analysis in aquatic environments. Polarization imaging addresses these issues by exploiting the polarization states of light, effectively reducing backscatter and enhancing image contrast. In this paper, we propose a polarization image fusion method guided by information entropy and a hierarchical-adaptive fusion strategy. Local information entropy is first employed to perform multiscale denoising on Degree of Linear Polarization (DOLP) images, enabling adaptive detail reconstruction while distinguishing texture from noise. Subsequently, a hierarchical fusion framework is applied: low-frequency components are enhanced through detail injection, while high-frequency components are fused using a structure-guided mechanism that leverages low-frequency gradient information to generate soft masks for phase-aligned detail integration and edge sharpening. Experiments conducted on self-collected underwater images, two public underwater datasets, and three general-scene datasets demonstrate that the proposed method improves objective metrics, including information entropy, average gradient, and edge strength. Subjective evaluations further confirm its effectiveness in preserving details and adapting to diverse scenes. Furthermore, rigorous ablation studies and runtime analyses demonstrate that the optimized framework achieves a highly favorable balance between robust, artifact-free detail enhancement and computational efficiency. The proposed approach provides a practical solution for underwater image enhancement, with potential applications in target detection and infrastructure inspection.

## 1. Introduction

High-quality underwater images are crucial for underwater exploration, nearshore port waste cleanup, as well as for inspecting the surface conditions and attached organisms of underwater infrastructure, such as bridge piers and pipelines. However, the turbidity of water directly affects imaging performance, often resulting in blurred images. This is mainly due to significant differences in the propagation characteristics of light in water compared to air, which can be categorized into three physical effects: light absorption causes color distortion in underwater images, typically giving them a bluish-green tone; forward and backward scattering leads to image blurring and reduced contrast, producing a hazy effect; and light refraction results in spatial perception distortion, making objects appear larger and closer than they actually are. These degradation issues, exacerbated by water turbidity, make the enhancement and restoration of underwater images a key challenge in advancing related applications.

To address these challenges, polarization imaging technology, which examines the polarization characteristics of light waves, has established itself as an effective approach for counteracting optical degradation in underwater environments. By offering informational support from a novel physical dimension, it opens up new possibilities for image recovery. In 2010, Li et al. [1] first demonstrated that polarization information could compensate for light-induced degradation effects, laying the groundwork for subsequent investigations. Huang et al. [2] further showed in 2016 that under complex underwater light fields where both object radiance and backscattering influence polarization, polarized images markedly enhance the discernibility of target details. In 2019, the team led by Ould Amer [3] proposed the adoption of a polarization imaging optical system to mitigate the impact of light diffusion on image acquisition. Advancing the field in 2023, Deng et al. [4] introduced a real-time underwater polarization imaging method that operates without background estimation. Their technique derives key parameters directly via Stokes vectors combined with frequency-domain low-pass filtering, making it suitable for scenarios involving moving targets and long-distance imaging. In 2024, Wu et al. [5] highlighted the pronounced backscatter suppression capability of polarization imaging and put forward a physics-driven deep learning descattering approach, which improved both interpretability and generalization in underwater polarization image processing. Noting in 2025 that underwater imaging methods based on the Angle of Polarization (AOP) are widely favored for their effective backscatter suppression, Li et al. accordingly developed a novel robust underwater polarization imaging method grounded in low-rank sparse decomposition of AOP [6]. Lin [7] similarly emphasized that polarization imaging serves as a key component of underwater imaging technology, as it suppresses backscattered light and thereby enhances overall image quality. Together, these studies substantiate that polarization imaging has become an indispensable technical means for tackling underwater optical degradation.

Although polarization imaging provides a valuable source of information, images relying on single-polarization information still cannot fully address all degradation issues. Therefore, it becomes essential to further integrate and enhance polarization information through image fusion methods. Existing approaches can be broadly classified into the following two categories.

### 1.1. Traditional Methods

Traditional underwater image enhancement methods primarily rely on transform domains and manually designed fusion rules. These can be broadly classified into three categories. The first category comprises methods based on multiscale transforms, including the wavelet transform [8], contourlet transform [9], non-subsampled shearlet transform (NSST) [10], and non-subsampled contourlet transform (NSCT) [11]. These techniques decompose an image into subbands of different scales and orientations, and perform fusion using rules such as coefficient weighting, region energy weighting, or adaptive fuzzy logic. While effective at preserving image details and separating approximate components, they generally entail high computational complexity. The second category includes approaches based on physical models and optimization constraints, which attempt to incorporate physical priors of underwater imaging. For instance, Fu et al. [12] employed a background region selection strategy combined with absorption compensation constraints for image restoration, whereas Zhang et al. [13] and Wang et al. [14], respectively, utilized multi-weight fusion or adaptive Gamma correction to improve visual naturalness and detail. These methods are highly dependent on optical priors and involve relatively high computational demands. The third category involves filtering and image fusion techniques, such as weighted guided filtering [15]. These are computationally simple but prone to issues such as local over-enhancement and detail distortion when processing complex underwater scenes. Conventional methods often struggle to balance detail enhancement, noise suppression, and contrast preservation in complex underwater environments, and their computational efficiency also remains a key bottleneck in practical applications.

### 1.2. Deep-Learning Methods

Leveraging its strong end-to-end feature learning capability, deep learning has significantly reduced reliance on handcrafted rules. In early studies, Hu et al. [16] pioneered the use of dense networks to learn the mapping relationship between object radiance and polarization information, establishing a learning-based approach to underwater polarization imaging. Subsequently, Generative Adversarial Networks (GANs) [17] were applied to learn the mapping from multi-polarization images to object radiance, thereby avoiding the dependence on physical priors required by traditional methods. More recent research has increasingly focused on fusion strategies and model robustness. For example, Guan et al. [18] employed a quality evaluator to generate quality maps for guiding the fusion process; Li et al. [19] integrated physical models with optimization algorithms for image restoration; and Ju et al. [20] introduced polarization-aware guidance and domain adaptation techniques to address data distribution shifts. While deep-learning methods have demonstrated promising performance in underwater image enhancement, they generally require large amounts of high-quality annotated data, and their generalization capability in turbid water environments remains an area for further improvement [19,20].

In addition to the aforementioned methods, recent research has further pushed the boundaries of underwater polarization imaging. For instance, polarimetric binocular three-dimensional imaging [21] has been developed to simultaneously recover depth information and enhance image quality in scattering media. Furthermore, generative approaches such as UPI2Diff [22], which utilizes a polarization-guided diffusion model, have demonstrated exceptional performance in restoration and noise suppression. These cutting-edge works represent the latest trends in the field and provide important context for the hierarchical framework proposed in this study.

In summary, while existing methods have achieved significant results in specific scenarios, they still exhibit noticeable limitations in enhancing details, suppressing noise, and maintaining contrast under complex imaging conditions. Accordingly, this paper proposes a multiscale polarization image denoising method based on local information entropy and a high-frequency fusion mechanism guided by low-frequency structural information. The approach aims to more effectively exploit the information from intensity images and polarization-degree images, thereby achieving high-quality enhancement of underwater images. It is worth noting that while our framework utilizes established foundational techniques—such as Contrast-Limited Adaptive Histogram Equalization (CLAHE) and Gaussian filters—these serve primarily as efficient, hardware-friendly substrates. The core novelty of our work lies in how we interpret and manipulate polarization information to guide the fusion process, rather than the basic spatial filters themselves. Furthermore, by deliberately choosing feed-forward spatial filtering over complex multiscale transforms (e.g., NSCT) or heavy deep neural networks, the proposed algorithm significantly reduces computational overhead. This design makes the entire framework highly conducive to parallel hardware acceleration in practical underwater engineering applications. The main contributions of this paper are as follows:

- This paper introduces a multiscale denoising approach for Degree of Linear Polarization (DOLP) images using information entropy. By analyzing the local entropy distribution of DOLP images, the method distinguishes textures from noise while maintaining a balance between noise removal and detail retention;
- This paper designs a low-frequency structure-guided high-frequency fusion mechanism, which dynamically generates fusion weights using the low-frequency gradient information of the intensity image. Adaptive fusion and enhancement of high-frequency details are realized through phase alignment and structure-aware soft masks;
- Experiments on both self-constructed and public datasets demonstrate that the proposed algorithm not only achieves superior objective fusion metrics but also significantly outperforms comparative algorithms in terms of subjective consistency.

## 2. Materials and Methods

**Figure 1:**
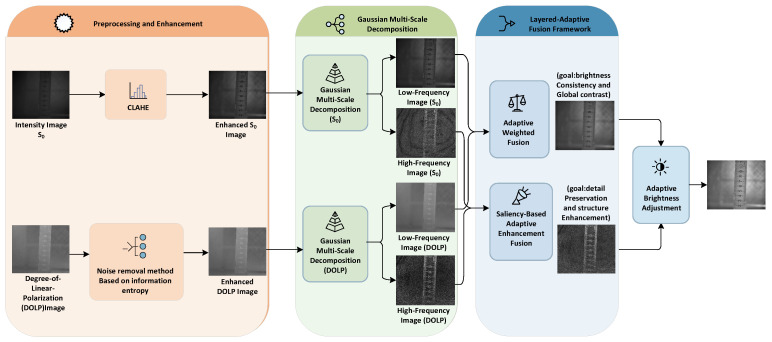
Framework of the Proposed Algorithm.

As systematically illustrated in the overall flowchart in Figure 1, the underwater polarization image fusion method proposed in this study consists of three key stages. First, the intensity image $S_{0}$ and the Degree of Linear Polarization (DOLP) image undergo preprocessing and enhancement, respectively: the $S_{0}$ image is processed using Contrast-Limited Adaptive Histogram Equalization (CLAHE) to enhance local contrast, while the DOLP image is denoised via a multiscale method based on local information entropy, which suppresses noise while preserving texture details. Second, Gaussian filtering is applied to decompose the preprocessed images into low-frequency and high-frequency components. Finally, a hierarchical-adaptive fusion framework is constructed to integrate the decomposed components: for the low-frequency part, detailed information extracted from the low-frequency component of the DOLP image is injected into the low-frequency component of the $S_{0}$ image to enhance overall structure and contrast; for the high-frequency part, a phase-aligned and structure-guided fusion strategy is introduced, which adaptively fuses multi-source high-frequency details based on low-frequency gradient information, thereby significantly improving edge sharpness. This section will separately detail the acquisition of polarization images and the specific steps of the fusion algorithm.

### 2.1. Acquisition of Polarization Images

**Figure 2:**
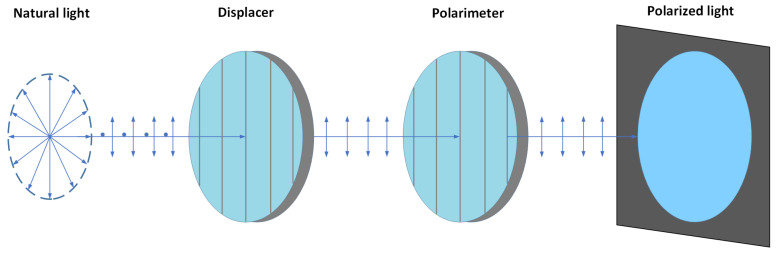
Schematic diagram of the polarization imaging principle. The radial arrows on the far left represent the omnidirectional electric field vibrations of unpolarized natural light. The horizontal single-headed arrows indicate the propagation direction of the light beam. The vertical double-headed arrows illustrate the specific linear polarization direction after the light passes through the polarizer.

The polarization information of a light field can be acquired by adjusting the transmission angle of a linear polarizer. When natural or partially polarized light passes through a linear polarizer oriented in a specific direction (such as horizontal or vertical), the component of its vibration direction that does not align with this orientation is filtered out, thereby obtaining linearly polarized light corresponding to that direction. By continuously rotating the angle of the polarizer, the transmittance of light waves with different vibration directions can be varied, allowing the acquisition of a series of polarization images at different polarization angles [23,24]. The principle of this process is illustrated in Figure 2: natural light, which originally vibrates in all planes perpendicular to the direction of propagation, is converted into linearly polarized light with a single vibration direction after passing through a polarizer. This linearly polarized light then passes through an analyzer. When the polarization direction of the analyzer is parallel to the vibration direction of the incident light, the transmittance of light intensity reaches its maximum [25].

After acquiring intensity images at four polarization angles ($0^{\circ }$, $45^{\circ }$, $90^{\circ }$, and $135^{\circ }$), the polarization characteristics of the light field can be quantitatively described using the Stokes vector. The Stokes vector consists of four variables ($S_{0}$, $S_{1}$, $S_{2}$, $S_{3}$), which can also be expressed as *I*, *Q*, *U*, and *V*. A commonly used definition is based on the four linearly polarized angle images *I*, *Q*, *U*, and *V*, along with the left- and right-handed circularly polarized light components IL and IR in the light wave. The specific definition is as follows [26]:

(1) $$S=\left[\begin{array}{c} S_{0}\\ S_{1}\\ S_{2}\\ S_{3} \end{array}\right]=\left[\begin{array}{c} I\\ Q\\ U\\ V \end{array}\right]=\left[\begin{array}{c} I_{0^{\circ }}+I_{90^{\circ }}\\ I_{0^{\circ }}-I_{90^{\circ }}\\ I_{45^{\circ }}-I_{135^{\circ }}\\ I_{L}-I_{R} \end{array}\right]$$

Here, $S_{0}$, $S_{1}$, $S_{2}$, and $S_{3}$ are the four parameters corresponding to the Stokes vector, which can fully characterize the intensity and polarization properties of a light beam. Based on the Stokes vector, the degree of polarization DOP of the polarized component of the light field can be calculated as:

(2) $$DOP=\frac{\sqrt{S_{1}^{2}+S_{2}^{2}+S_{3}^{2}}}{S_{0}}$$

In practical applications, the circular polarization component $S_{3}$ is typically weak, and the primary consideration is the influence of linearly polarized light. In such cases, the degree of linear polarization DOLP can be defined as:

(3) $$DOLP=\frac{\sqrt{S_{1}^{2}+S_{2}^{2}}}{S_{0}}$$

### 2.2. Fusion Algorithm

#### 2.2.1. Preprocessing of Intensity Image: CLAHE

Intensity images contain detailed information missing in polarization images, such as boundaries of specific materials and surface defects. To ensure high-quality input for subsequent fusion, the intensity image $S_{0}$ is first enhanced for contrast. Traditional histogram equalization methods can improve contrast but often lead to over-enhancement or noise diffusion, making them less suitable for natural scenes with spatially varying brightness. Therefore, this study employs Contrast-Limited Adaptive Histogram Equalization CLAHE to enhance the $S_{0}$ image. The core principle of CLAHE is to perform histogram equalization separately within local regions and clip the histogram of each sub-block to suppress excessive noise amplification. The core process of CLAHE mainly consists of the following steps: sub-block partitioning and histogram statistics, histogram clipping and redistribution, local equalization, and cross-block interpolation reconstruction [27].

From a mathematical perspective, the intensity of contrast enhancement is proportional to the slope of the cumulative distribution function (CDF), which depends on the height of the histogram bins. In underwater images, concentrated background noise often forms sharp peaks in the histogram. By clipping these peaks to a predefined threshold, CLAHE effectively constrains the maximum slope of the transformation function, thereby strictly suppressing excessive amplification of noise contrast.

#### 2.2.2. Polarization Image Filtering: IEB-MSD

Underwater polarization images can capture texture and detail information that is difficult to directly characterize through traditional intensity imaging. However, the abundant suspended particles and dissolved substances in water cause scattering and absorption effects, generating forward and backward scattered light with polarization properties, which introduces significant noise. Moreover, the calculation of the DOLP image involves nonlinear operations on multiple polarization-direction images, making it highly sensitive to such noise. As the DOLP image serves as crucial information for subsequent underwater image fusion, severe noise contamination can degrade the effectiveness and robustness of subsequent processing algorithms. Traditional edge-aware methods based on gradients or edge strength [28] often misclassify random noise as genuine edges in the DOLP image, further compromising the robustness of the algorithm.

**Figure 3:**
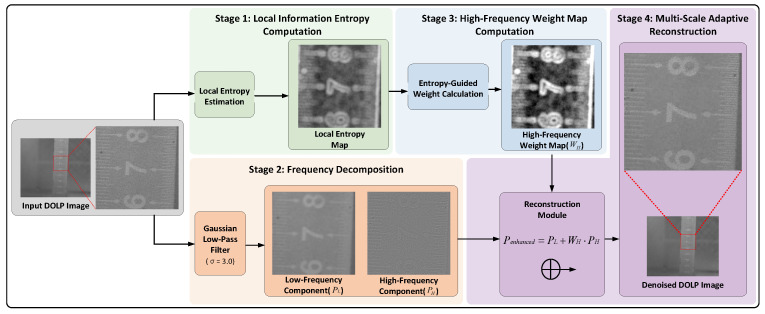
Flowchart of Polarization Image Filtering.

To address the issues mentioned above, this paper proposes an information entropy-based multiscale denoising method (IEB-MSD) for DOLP images. This method distinguishes between texture details and noise in DOLP image using local information entropy values and decomposes the input DOLP image into high-frequency and low-frequency components using a Gaussian low-pass filter. Based on the local information entropy value of each pixel, a high-frequency reconstruction weight map is computed for each sub-block. Finally, the high-frequency weight map guides the reconstruction of both the high-frequency and low-frequency components of the DOLP image. The overall flowchart of the proposed multiscale denoising algorithm for DOLP image is shown in Figure 3, which primarily consists of four parts: local information entropy calculation, high- and low-frequency decomposition, high-frequency weight map computation, and multiscale reconstruction.

The first step of the multiscale denoising algorithm for the DOLP image is to compute the local information entropy of the DOLP image. Let the input DOLP image be denoted as P(x,y), with its total number of gray levels represented by *N*, which is set to 256 in this paper. First, within a local neighborhood $\Omega_{x,y}$ centered at pixel (x,y) with a radius of *r*, the probability distribution of gray levels is calculated as shown in Equation (4), where *n* represents the gray level. The local polarization information entropy for this pixel is then defined by Equation (5). Subsequently, the local polarization information entropy of each pixel is normalized as given in Equation (6). The normalized result satisfies $E^{\mathrm{norm}}\left(x,y\right)\in \left[0,1\right]$ and can be used to characterize the relative richness of polarization information at that pixel. This value will be employed in subsequent steps to compute the reconstruction weight map for the high-frequency components.

(4) $$p_{n}\left(x,y\right)=\frac{1}{|\Omega_{x,y}|}\sum_{\left(u,v\right)\in \Omega_{x,y}}\delta \left(P\left(u,v\right)=n\right)$$

(5) $$E\left(x,y\right)=-\sum_{n=0}^{N-1}p_{n}\left(x,y\right)\log_{2}p_{n}\left(x,y\right)$$

(6) $$E^{\mathrm{norm}}\left(x,y\right)=\frac{E\left(x,y\right)-\min\left(E(x,y)\right)}{\max\left(E(x,y)\right)-\min\left(E(x,y)\right)}$$

The core novelty of the proposed IEB-MSD lies in its shift from traditional spatial-gradient perspectives to an information-theoretic approach. Conventional edge-aware filters (e.g., bilateral or guided filters) rely heavily on pixel intensity differences; consequently, they often misclassify severe, high-contrast polarization speckle noise as structural edges, failing to denoise effectively. In contrast, our method utilizes normalized local entropy ($E^{\mathrm{norm}}$) as a robust discriminator. By mathematically quantifying the true informational richness of a local region, IEB-MSD can precisely isolate stochastic granular noise (which presents as abnormal entropy variations) from genuine physical textures, achieving optimal detail preservation in highly degraded underwater polarization fields.

In the second step, a Gaussian low-pass filter is applied to decompose the input DOLP image P(x,y) in the frequency domain, separating it into a low-frequency component $P_{L}\left(x,y\right)$ and a high-frequency component $P_{H}\left(x,y\right)$. Specifically, the low-frequency component is obtained by convolving the input image P(x,y) with a Gaussian kernel, as expressed in Equation (7).

(7) $$P_{L}\left(x,y\right)=\sum_{i=-k}^{k}\sum_{j=-k}^{k}P\left(x+i,y+j\right)\cdot G\left(i,j,\sigma \right)$$

(8) $$P_{H}\left(x,y\right)=P\left(x,y\right)-P_{L}\left(x,y\right)$$

(9) $$G\left(x,y,\sigma \right)=\frac{1}{2\pi \sigma^{2}}e^{-\frac{x^{2}+y^{2}}{2\sigma^{2}}}$$

Here, G(x,y,σ) denotes a two-dimensional Gaussian function with standard deviation σ. In this study, the standard deviation of the Gaussian low-pass filter is empirically set to σ=2.0 (with a kernel size of 30 to prevent truncation artifacts). Mathematically, the effective −3 dB (half-power) cutoff frequency $f_{c}$ of a Gaussian filter is inversely proportional to its standard deviation, determined by solving $|H\left(f_{c}\right)|=\exp\left(-2\pi^{2}\sigma^{2}f_{c}^{2}\right)=1/\sqrt{2}$. This yields the relationship $f_{c}=\sqrt{\ln2}/\left(2\pi \sigma \right)$. Setting σ=2.0 corresponds to a cutoff frequency of approximately $f_{c}\approx 0.0663$ cycles/pixel. This specific parameter is selected because underwater polarization speckle noise typically manifests as extreme, isolated pixel-level fluctuations residing in the higher frequency spectrum (variations smaller than a 2-to-3-pixel radius). This cutoff frequency optimally isolates the granular noise into the high-frequency component $P_{H}$, while strictly preserving the fundamental structural contours and illumination gradients in the low-frequency component $P_{L}$.

Before reconstruction, it is necessary to determine the reconstruction weight map for the high-frequency components. The method adopted in this algorithm is to guide the calculation of the weight map based on the normalized information entropy $E^{\mathrm{norm}}\left(x,y\right)$ of each pixel. Regions with high entropy, which correspond to texture details, are assigned larger weights, while regions with low entropy, such as flat areas or noise, are assigned smaller weights. A value closer to 1 indicates richer texture details in the region, whereas a value closer to 0 suggests that the region tends to be flat or predominantly composed of background noise. Based on the normalized entropy values, an adaptive weight mapping function is constructed. The purpose of this function is to assign corresponding denoising control weights to pixels of varying complexity. In this paper, a linear mapping strategy is employed, with the calculation formula given in Equation (10). Here, W(x,y) represents the adaptive weight coefficient for pixel(x, y), while $W_{\min}$ and $W_{\max}$ are preset upper and lower weight thresholds, respectively, used to define the processing boundaries of the algorithm between flat noisy regions and textured regions.

(10) $$W\left(x,y\right)=W_{\min}+E^{\mathrm{norm}}\left(x,y\right)\cdot \left(W_{\max}-W_{\min}\right)$$

Finally, the adaptive weight coefficient W(x,y) is utilized as the high-frequency weight $W_{\mathrm{high}}\left(x,y\right)$ during reconstruction. The low-frequency and high-frequency components are recombined using a weighted reconstruction approach, as expressed in Equation (11).

(11) $$P_{\mathrm{enhanced}}\left(x,y\right)=W_{\mathrm{high}}\left(x,y\right)\cdot P_{H}\left(x,y\right)+W_{\mathrm{low}}\left(x,y\right)\cdot P_{L}\left(x,y\right)$$

Here, since the low-frequency component $P_{L}\left(x,y\right)$ primarily contains the overall structure and slowly varying brightness information of the image, the low-frequency weight $W_{\mathrm{low}}\left(x,y\right)$ is consistently set to 1 during reconstruction, indicating that the low-frequency component is completely preserved. $P_{\mathrm{enhanced}}\left(x,y\right)$ represents the enhanced and denoised DOLP image.

#### 2.2.3. Low- and High-Frequency Fusion Algorithm

The core objective of decomposing an image into low-frequency and high-frequency components is to separate the global structural information from local details, textures, and noise, thereby enabling targeted processing of different components [29]. Traditional image decomposition methods include linear/nonlinear filtering approaches (such as bilateral filtering [30], guided filtering [31], Gaussian filtering, etc.), multiscale transform methods (e.g., NSCT, Wavelet, Curvelet [32], etc.), and frequency-based Fourier decomposition [33,34] methods. Methods like NSCT possess translation invariance and offer good reconstruction capability, but they involve complex computations and high resource consumption. Fourier-based frequency decomposition, while effective in analyzing spectral information, has limited ability to handle local spatial information. Therefore, to balance computational efficiency and implementation complexity while ensuring smooth extraction of low-frequency information, this study adopts the same Gaussian filtering method described in polarization image filtering as the decomposition approach. It can simply and efficiently separate the low-frequency structure and high-frequency details of the image, and is easy to apply in subsequent processing and fusion algorithms. In this paper, a Gaussian kernel with a standard deviation of σ=2.0 is used to decompose the image into a low-frequency component $L_{\mathrm{low}}\left(x,y\right)$ and a high-frequency component $H_{\mathrm{high}}\left(x,y\right)$. The low-frequency component is obtained through convolution, while the high-frequency component is calculated as the difference between the original image and the low-frequency component. The value of *k*, representing the spatial radius of the Gaussian convolution kernel that determines a total window size of 2k+1, is set to 3σ to ensure coverage of the Gaussian function’s main energy, thereby preventing truncation artifacts according to the three-sigma rule.

The specific steps of the fusion algorithm are as follows:

Step 1: Low-frequency Fusion Rule: To effectively fuse the low-frequency information from the intensity image $S_{0}$ and the Degree of Linear Polarization (DOLP) image, this paper proposes a detail injection-based fusion method. This approach takes the low-frequency component $L_{1}$ of the intensity image as the foundation and achieves significant enhancement of the overall contrast and texture information in the fused image by extracting and integrating the structural details from the low-frequency component $L_{2}$ of the DOLP image. The specific steps are as follows:

1. Extraction of detail components. The DOLP image contains richer edge and structural information, and its low-frequency component $L_{2}$ is abundant in structural details. To separate such information, a second Gaussian filtering is first applied to $L_{2}$ to obtain its very-low-frequency background estimate $L_{2,\mathrm{blur}}$. The filtering employs a larger kernel size *K* and standard deviation σ, aiming to effectively filter out mid- to high-frequency details while preserving the globally smooth background. The filtering calculation can be expressed by Equation (12), where ∗ denotes the convolution operation. Subsequently, the detail component *D* in $L_{2}$ is extracted through a difference operation, as described in Equation (13).
  (12) $$L_{2,\mathrm{blur}}=G_{\sigma =7,K=21}*L_{2}$$
  (13) $$D=L_{2}-L_{2,\mathrm{blur}}$$
2. Detail enhancement and fusion. The directly extracted detail components *D* may contain noise and exhibit limited contrast. To enhance the detail components and suppress potential noise, linear enhancement is applied to the detail components:

(14) $$D_{\mathrm{enh}}=\alpha \cdot D$$

Here, the enhancement coefficient α is set to 0.5, achieving a good balance between improving detail saliency and controlling noise amplification. Subsequently, the enhanced detail components $D_{\mathrm{enh}}$ are added to the low-frequency component $L_{1}$ of the intensity image to obtain the fused low-frequency component $L_{\mathrm{fused}}$.

(15) $$L_{\mathrm{fused}}=L_{1}+D_{\mathrm{enh}}$$

**Figure 4:**
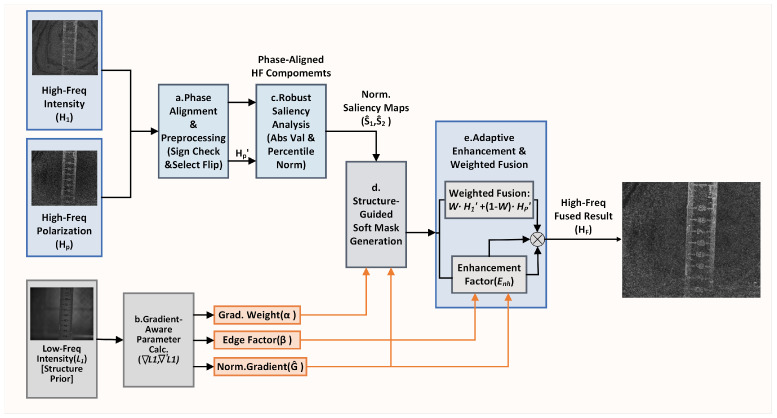
Flowchart of High-Frequency Image Fusion.

Step 2: High-frequency Fusion Rule: The algorithm adopts a cross-band fusion strategy termed “structure-guided detail enhancement.” This strategy innovatively incorporates a phase alignment preprocessing mechanism, synergistically integrates gradient-aware dynamic parameter adjustment, and employs a robust saliency assessment method. It is designed to precisely resolve conflicts among multimodal high-frequency information and optimize the selection and enhancement of details based on the intrinsic structure of the images. The core idea is to utilize the structural information carried by the low-frequency components as prior knowledge to dynamically guide the fusion decisions for high-frequency details. This ensures that effective textures and edges from the source images are fully preserved, while producing a clear, natural, and visually enhanced fused result. The specific workflow is illustrated in Figure 4.

1. Phase Alignment and Preprocessing. Let $H_{1}$ and $H_{2}$ represent the extracted high-frequency components of the intensity image and the DOLP image, respectively. Due to differences in the response of light intensity and polarization characteristics, intensity images and DOLP images often exhibit opposite contrast in edge regions. To address this issue, a selective phase inversion is performed. If the structural edge gradients of the two high-frequency components have opposite signs (i.e., $H_{1}⊙H_{2}<0$), the phase of $H_{2}$ is inverted to align with $H_{1}$. The aligned high-frequency component $H_{2}^{\prime }$ is computed as:
  (16) $$H_{2}^{\prime }=\left\{\begin{array}{cc} -H_{2}, & \mathrm{if}\;H_{1}⊙H_{2}<0\\ H_{2}, & \mathrm{otherwise} \end{array}\right.$$
2. Gradient-Aware Adaptive Parameter Calculation. Structural feature analysis is conducted on the low-frequency component of the intensity image to dynamically compute the gradient-aware weight factor α, the edge enhancement factor β, and the normalized gradient map $G_{\mathrm{norm}}$, as detailed in Equations (17)–(19). Here, the gradient-aware weight factor α reflects the importance of local structures in the image: regions with larger gradient values yield higher α, indicating richer structural information that should receive greater attention during fusion. The edge enhancement factor β quantifies the sharpness of edges: larger second-order gradient values correspond to sharper edges, which require stronger enhancement. The normalized gradient map $G_{\mathrm{norm}}$ linearly maps the first-order gradient magnitude to the interval [0,1], providing a standardized reference for subsequent weighted fusion and eliminating differences in gradient magnitude across images. In the equations, ∇ and $\nabla^{2}$, respectively, denote the gradient and Laplacian operators; ∥·∥ represents the gradient magnitude computation; and $f_{1}$, $f_{2}$ are nonlinear mapping functions used to convert gradient magnitude into weight factors and to quantify edge information as enhancement coefficients, respectively.
  (17) $$\alpha =f_{1}\left(∥\nabla L_{\mathrm{low}}∥\right)$$
  (18) $$\beta =f_{2}\left(∥\nabla^{2}L_{\mathrm{low}}∥\right)$$
  (19) $$G_{\mathrm{norm}}=\frac{∥\nabla L_{\mathrm{low}}∥-\min\left(∥\nabla L_{\mathrm{low}}∥\right)}{\max\left(∥\nabla L_{\mathrm{low}}∥\right)-\min\left(∥\nabla L_{\mathrm{low}}∥\right)}$$
3. Robust Saliency Analysis. To quantify the importance of high-frequency details, the absolute values of the phase-aligned high-frequency coefficients are computed to obtain the initial saliency maps, denoted as $S_{k}^{\mathrm{raw}}$. To prevent a small number of extreme noise outliers from dominating the normalization process, we introduce a robust normalization method instead of the standard min-max scaling. Specifically, we calculate the 99.5-th percentile of $S_{k}^{\mathrm{raw}}$, denoted as $S_{\max}^{99.5}$, and its minimum value, denoted as $S_{\min}$. The normalized saliency map $S_{k}$ is then computed as:
  (20) $$S_{k}\left(x,y\right)=\min\left(1,\frac{S_{k}^{\mathrm{raw}}\left(x,y\right)-S_{\min}}{S_{\max}^{99.5}-S_{\min}}\right)$$
  This simple truncation ensures that the top 0.5% of extreme pixel values (typically sharp noise) are clamped to 1, providing a highly stable and robust saliency representation for subsequent fusion.
4. Generation of Structure-Guided Soft Mask. By integrating the gradient-aware weight α, the normalized gradient $G_{\mathrm{norm}}$, and the saliency values $S_{1}$ and $S_{2}$, a soft mask weight map $M_{\mathrm{soft}}$ is generated through nonlinear mapping. The soft mask weight map $M_{\mathrm{soft}}$ comprehensively considers both saliency differences and gradient structural information. When $S_{1}$ is significantly greater than $S_{2}$, $M_{\mathrm{soft}}$ approaches 1, giving priority to the detail information from $H_{1}$; when $S_{2}$ is significantly greater than $S_{1}$, $M_{\mathrm{soft}}$ approaches 0, favoring the detail information from $H_{2}$; when the two are comparable, the decision is adjusted using the gradient-aware weight α and the normalized gradient $G_{\mathrm{norm}}$, tending to preserve the information source with clearer structure in regions where the gradient is prominent.
  (21) $$M_{\mathrm{soft}}=\frac{S_{1}+\alpha \cdot G_{\mathrm{norm}}}{S_{1}+S_{2}+\alpha \cdot 2G_{\mathrm{norm}}}$$
  The distinct novelty of this high-frequency fusion mechanism is its cross-band guidance strategy via the structure-aware soft mask ($M_{\mathrm{soft}}$). Traditional high-frequency fusion rules, such as the widely used ‘choose-max absolute’ or Principal Component Analysis (PCA), treat high-frequency subbands in isolation. This blind integration frequently leads to unnatural edge halos, contrast reversal, or the introduction of artifacts from unsuppressed noise. Our approach fundamentally overcomes this by dynamically bridging the frequency bands. By leveraging the pristine structural priors inherent in the low-frequency intensity gradients ($G_{\mathrm{norm}}$) to dictate the high-frequency fusion weights, we ensure that the injection of polarization details strictly adheres to the authentic physical boundaries of the targets, resulting in mathematically aligned and strictly artifact-free edge sharpening.
5. Adaptive Enhancement and Fusion. First, the enhancement factor $E_{\mathrm{enhance}}$ is dynamically calculated according to Equation (22), using the edge enhancement factor β and the normalized gradient $G_{\mathrm{norm}}$. The enhancement factor adaptively adjusts based on gradient magnitude: higher $G_{\mathrm{norm}}$ values correspond to stronger enhancement. Simultaneously, the β factor further refines the enhancement by incorporating edge sharpness, resulting in intensified sharpening in well-defined edge regions while maintaining stability in flat areas to prevent noise amplification. The resulting $H_{\mathrm{fused}}$, generated through soft-mask weighting, effectively improves contrast and clarity in structural regions.
  (22) $$E_{\mathrm{enhance}}=1+\beta \cdot G_{\mathrm{norm}}$$
  (23) $$H_{\mathrm{fused}}=E_{\mathrm{enhance}}\cdot \left[M_{\mathrm{soft}}⊙H_{1}+\left(1-M_{\mathrm{soft}}\right)⊙H_{2}\right]$$

Step 3: The final fused image $I_{\mathrm{fused}}$ is obtained by summing the low-frequency fused image $L_{\mathrm{fused}}$ and the high-frequency fused image $H_{\mathrm{fused}}$. Since the denoising process for polarization images tends to reduce the overall local brightness, an adaptive brightness adjustment is applied to the fused image to enhance overall contrast. Specifically, the ratio of the current average brightness μ of the image to the target mid-level brightness is calculated as the adjustment factor, which is then capped at 1.5. A uniform linear scaling is subsequently applied to all pixels in the image. This approach gently adjusts the image brightness to a visually comfortable range while avoiding over-enhancement that could lead to image distortion.

(24) $$I_{\mathrm{fused}}=L_{\mathrm{fused}}+H_{\mathrm{fused}}$$

(25) $$I_{\mathrm{enhanced}}\left(x,y\right)=\mathrm{clip}\left(I_{\mathrm{fused}}\left(x,y\right)\cdot \min\left(1.5,\frac{0.5}{\mu }\right),\;0,\;1\right)$$

## 3. Results

To systematically evaluate the performance of the fusion algorithm proposed in this paper, this chapter conducts validation from three aspects: experimental design, visual comparison, and quantitative analysis. Section 3.1 first introduces the hardware platform, dataset composition, and benchmark methods used in the experiments. Next, Section 3.2 presents underwater imaging experiments on physical objects such as steel rulers, stones, and plastic bottles, comparing the proposed algorithm with eight mainstream methods through both visual assessment and objective metrics. Finally, tests conducted on two publicly available underwater datasets further verify the comprehensive performance and advantages of the proposed method.

### 3.1. Experimental Setup

This experiment utilizes a polarization camera, the MER2-550-POL, which is equipped with a global exposure Sony IMX264MZR CMOS sensor chip. The camera is capable of simultaneously capturing polarization images at angles of 0°, 45°, 90°, and 135°, with a resolution of 2448 × 2048 pixels. It employs a USB3.0 interface for image data transfer, and the third party software Galaxy SDK is used to configure the camera and acquire the corresponding four angle images.

For the validation of the fusion method proposed in this paper, we selected self-collected images, two public underwater image datasets: U2PNet [35], UPBD [36]—as the data sources. Meanwhile, eight existing methods were chosen for comparison with our approach. The eight methods are listed in Table 1, among which method 1 and method 2 are polarization image fusion methods designed for general scenes; method 3 is an underwater polarization image fusion method; and methods 4, 5, and 6 are underwater visible-light enhancement methods; methods 7 and 8 (PAPIF and CPIFuse) are state-of-the-art deep-learning-based polarization image fusion networks, included to comprehensively evaluate our method against contemporary data-driven architectures. This study selects five key metrics from four dimensions—information content, clarity, structure, and fidelity—based on references [37,38,39,40]. Specifically, entropy (EN) evaluates the information richness of the fused image; standard deviation (SD) measures its overall contrast; average gradient (AG) and edge intensity (EI) quantify detail sharpness and edge-feature preservation, respectively; while spatial frequency (SF) reflects the overall detail activity of the image.

To ensure maximum methodological transparency and facilitate the reproducibility of our proposed framework, the determination strategies for all key hyperparameters are systematically summarized in Table 2. Rather than relying on rigid empirical constants, our framework determines these parameters either through strict mathematical derivations (e.g., the 3σ rule) or comprehensive quantitative ablation studies. This adaptive and rigorously verified parameter selection mechanism ensures an optimal balance between detail enhancement and noise suppression across diverse underwater scenarios.

### 3.2. Experimental Results and Analysis

In response to issues such as low contrast and blurred details in underwater images caused by scattering, refraction, and other effects, this study selected objects, including a steel ruler, stones, plastic bottles, agate, and algae balls, to represent metal materials, rough minerals, plastic waste, ceramics, and biological attachments, respectively, for experimental validation. In each scenario, (a,b) denote the intensity image $S_{0}$ and the degree of linear polarization (DOLP) image, (c–h) correspond to the fused images obtained by the six reference fusion algorithms, (i,j) correspond to the newly added deep-learning baselines (PAPIF and CPIFuse), and (k) represents the fused image generated by the proposed algorithm in this paper.

**Figure 5:**
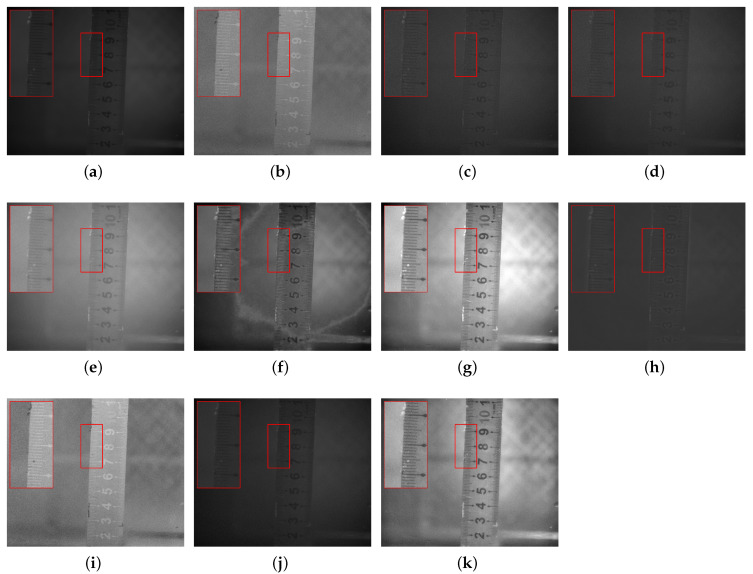
Scene 1: Underwater Steel Ruler. (**a**) $S_{0}$. (**b**) DOLP. (**c**–**j**) Fusion images generated by methods 1 to 8, respectively. (**k**) Fused image from the proposed method. The red bounding boxes and the corresponding zoomed-in patches are added to highlight the detailed performance in texture recovery and noise suppression.

In Figure 5, the intensity image appears overall too dark, with edge details being indistinct. Fusing the polarization-degree image yields a result with more balanced detail and brightness. Images (c,d,h) produced are too dark overall, failing to capture fine details; image (f) exhibits severe distortion; the quality of image (e) degrades noticeably, appearing blurred; while image (g) enhances brightness but tends to over-amplify background noise. Regarding the deep-learning methods, PAPIF (i) extracts the salient polarization edges but inadvertently amplifies the background granular noise, exhibiting typical domain-shift artifacts in degraded underwater scenarios. CPIFuse (j) produces a visually soft result, failing to effectively transfer the high-frequency polarization textures of the steel ruler, which leads to blurred edge details. In contrast, the fused image generated by the proposed algorithm (k) achieves the most favorable balance. It strictly preserves the sharp polarization characteristics of the steel ruler while effectively suppressing background speckle noise, yielding clear, natural, and artifact-free details.

**Figure 6:**
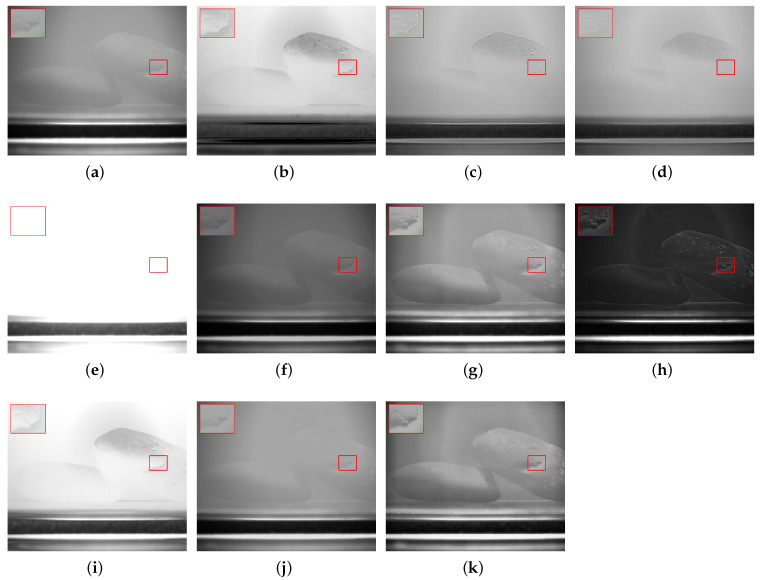
Scene 2: Underwater Stones. (**a**) $S_{0}$. (**b**) DOLP. (**c**–**j**) Fusion images generated by methods 1 to 8, respectively. (**k**) Fused image from the proposed method. The red bounding boxes and the corresponding zoomed-in patches are added to highlight the detailed performance in texture recovery and noise suppression.

In Figure 6, images (c,d,h) suffer from overall low contrast, blurred object edges, and substantial loss of detail. Image (e) exhibits severe processing artifacts along with local overexposure. Image (f) introduces noticeable structural distortion during the fusion process. Image (g) improves the contrast of the target region to some extent, but the background still appears relatively flat. Regarding the deep-learning approaches, PAPIF (i) incorrectly treats the ubiquitous underwater speckle noise as salient features, leading to severe granular noise amplification across both the stones and the background. CPIFuse (j), on the other hand, exhibits an over-smoothing effect, losing the critical high-frequency crack details distinctly captured in the DOLP image. In contrast, the fused image generated by the proposed algorithm (k) achieves a more optimal balance among luminance distribution, detail clarity, and structural integrity, effectively highlighting the surface textures and cracks of the stones while maintaining a clean, noise-suppressed background.

**Figure 7:**
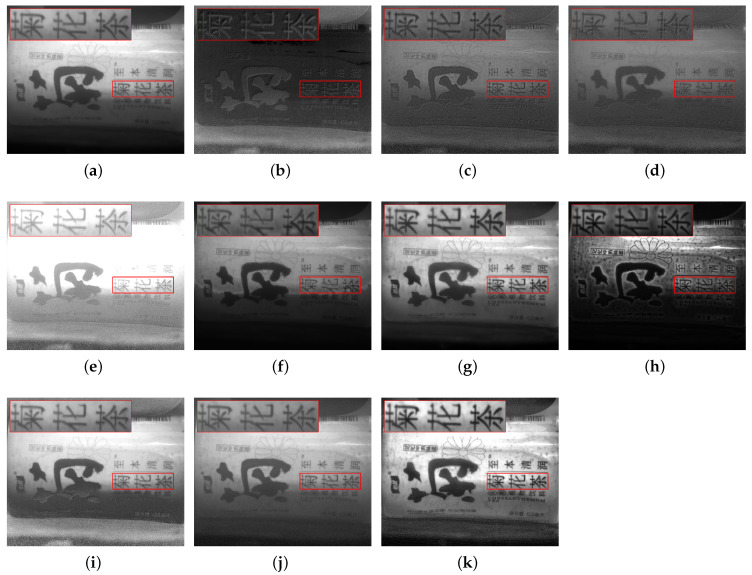
Scene 3: Underwater Plastic Bottle. (**a**) $S_{0}$. (**b**) DOLP. (**c**–**j**) Fusion images generated by methods 1 to 8, respectively. (**k**) Fused image from the proposed method. The red bounding boxes and the corresponding zoomed-in patches are added to highlight the detailed performance in texture recovery and noise suppression.

In Figure 7, images (c,d) appear overall grayish, with limited contrast improvement after fusion; the text edges on the bottle body are blurred, and the detail hierarchy is unclear. Image (e) exhibits noticeable over-enhancement of brightness, leading to saturation in highlight areas and partial loss of textual information. Image (f) is generally dark; although it suppresses some background interference, the information in the target region is not fully retained. Image (g) enhances the surface texture and text legibility of the plastic bottle to a certain extent, but is still accompanied by strong background noise and local luminance inhomogeneity. Image (h) strongly reinforces edge information, but simultaneously amplifies noise and artifacts. When evaluating the deep-learning models, PAPIF (i) fails to distinguish between salient text features and underwater scattering, resulting in a severe amplification of granular speckle noise across the entire image. CPIFuse (j) effectively suppresses noise but excessively smooths the image, causing the high-frequency text details on the plastic bottle to become severely blurred and illegible. In contrast, the fused image generated by the proposed algorithm (k) presents the text and pattern structure on the bottle surface more clearly while effectively suppressing underwater scattering and reflection interference, perfectly avoiding both the noise amplification and the over-smoothing artifacts seen in the data-driven approaches.

**Figure 8:**
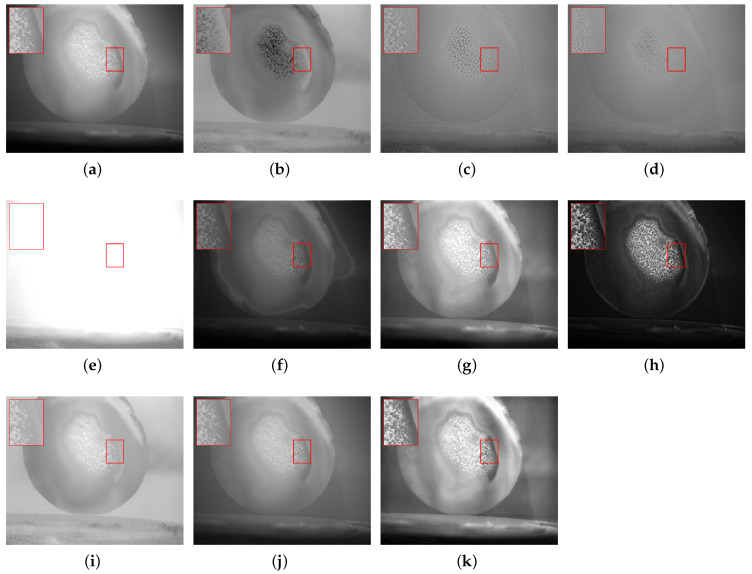
Scene 4: Underwater Agate Slice. (**a**) $S_{0}$. (**b**) DOLP. (**c**–**j**) Fusion images generated by methods 1 to 8, respectively. (**k**) Fused image from the proposed method. The red bounding boxes and the corresponding zoomed-in patches are added to highlight the detailed performance in texture recovery and noise suppression.

In Figure 8, images (c,d,h) yield results with overall insufficient contrast, a dark appearance, and severe loss of detail. Image (e) suffers from significant degradation in quality after processing, with underexposure making the image difficult to discern. Image (f) introduces noticeable geometric distortion or structural deformation during the fusion process, compromising the accuracy of the object’s form. Image (g) achieves relatively better processing results among the traditional methods by preserving some polarization characteristics of the target. However, when examining the deep-learning baselines, PAPIF (i) introduces prominent granular noise into the background, failing to cleanly separate the target’s textures from water scattering. CPIFuse (j) suffers from a severe loss of global contrast, resulting in a completely washed-out appearance that severely obscures the fine concentric banding details of the agate slice. In contrast, the fused image generated by the proposed algorithm (k) achieves the most optimal visual quality. It preserves the polarization characteristics of the target highly effectively, revealing the crisp concentric textures of the agate slice while maintaining a clean, high-contrast background.

**Figure 9:**
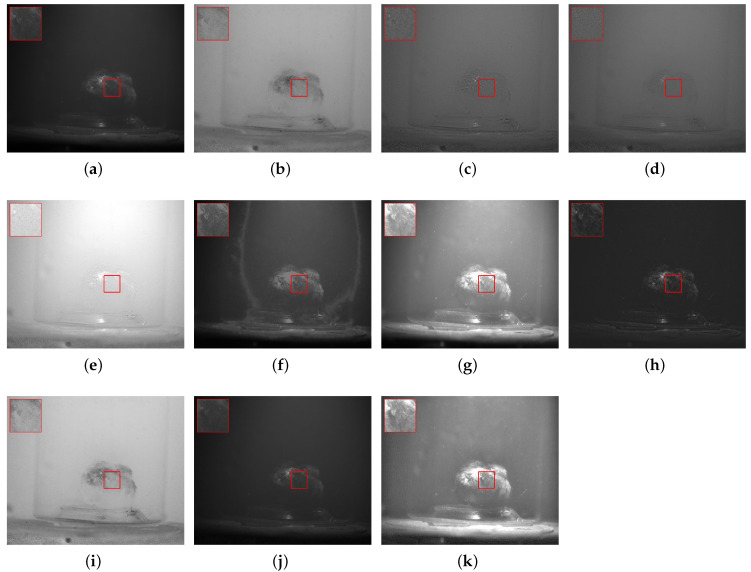
Scene 5: Underwater Algae Ball. (**a**) $S_{0}$. (**b**) DOLP. (**c**–**j**) Fusion images generated by methods 1 to 8, respectively. (**k**) Fused image from the proposed method. The red bounding boxes and the corresponding zoomed-in patches are added to highlight the detailed performance in texture recovery and noise suppression.

In Figure 9, images (c,d) appear overall dark, with limited contrast improvement after fusion; the edge and internal texture information of the algae ball are not effectively restored. Image (e) exhibits obvious over-enhancement, with excessively high brightness in the background area. Image (f) introduces noticeable artifacts and structural distortion near the target edges. Image (g) enhances the brightness and contrast of the algae ball to some extent, but strong scattering interference remains in the background. Image (h) is generally too dark, causing the target information to be submerged again in the dark background. As for the deep-learning models, PAPIF (i) incorrectly amplifies background scattering as structural features, resulting in a heavily noise-contaminated image that severely degrades the overall visual experience. CPIFuse (j) produces a highly smoothed and excessively darkened result, completely failing to restore the fine, hairy surface textures inherent to the algae ball. In contrast, the fused image generated by the proposed algorithm (k) clearly presents the contour structure and surface texture characteristics of the algae ball, achieving the highest visual fidelity while maintaining a remarkably clean background.

From a visual perspective, the proposed method achieves more stable fusion performance under complex underwater imaging conditions. While avoiding issues such as excessive darkness, overexposure, and structural distortion, the method effectively enhances the contrast and detail clarity of target regions, and significantly suppresses underwater scattering and fusion artifacts. Furthermore, the method preserves the polarization characteristics of targets effectively, making object contours, textures, and textual information more distinct and recognizable. Overall, the visual quality outperforms that of the comparative methods.

To objectively evaluate the image quality obtained by several algorithms, Table 3, Table 4, Table 5, Table 6 and Table 7 present the objective evaluation metrics for the five different scenes, respectively.

As can be seen from the above tables, the fusion images obtained by our proposed algorithm rank first or second in most evaluation metrics. Meanwhile, to validate the accuracy and applicability of our algorithm for enhanced images in underwater scenarios, we conducted experiments using two publicly available underwater datasets from the Internet, U2PNet and UPBD, as detailed in Table 8 and Table 9. It can be observed that when compared with underwater algorithms 3 to 8, our algorithm consistently achieves superior metrics. The excessively high AG and SF values obtained by algorithms 1 and 2 are attributed to the severe distortion in their resulting images, which leads to amplified edges.

**Figure 10:**
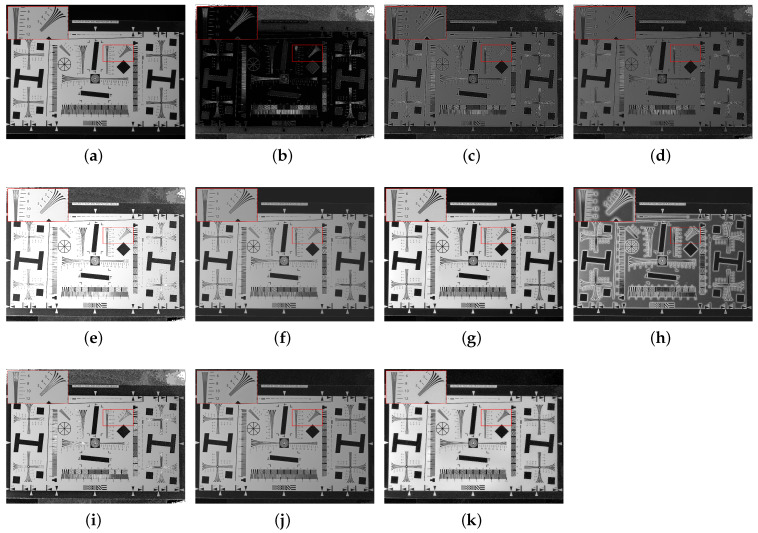
Scene 6: Underwater resolution test card. (**a**) $S_{0}$. (**b**) DOLP. (**c**–**j**) Fusion images generated by methods 1 to 8, respectively. (**k**) Fused image from the proposed method. The red bounding boxes and the corresponding zoomed-in patches are added to highlight the detailed performance in texture recovery and noise suppression.

To further objectively validate the algorithm’s performance in detail recovery and noise suppression, we evaluated a standard resolution chart scene from the UPBD dataset (Figure 10). Analysis of the source images reveals their inherent limitations: the low contrast of the intensity image ($S_{0}$) causes dense micro-scales and high-frequency lines to become blurred; while the degree of linear polarization (DOLP) image—despite possessing specific physical edge characteristics—is severely degraded by background speckle noise, which completely submerges effective structural information. When observing the baseline methods, none achieved a satisfactory balance. The fusion results of Algorithms 1 and 2 are overly dark and lack sharpness. Algorithms 3 and 4 performed poorly in noise control; the former amplified background noise into a grainy texture, while the latter lost high-frequency details due to over-smoothing. Furthermore, Algorithm 5 suffers from local overexposure, causing bright lines to blend together, and Algorithm 6 introduced highly unnatural halo artifacts and structural distortions around geometric blocks. Similarly, the deep-learning baselines failed these extremely high-frequency challenges. PAPIF (i) misinterprets speckle noise as features, destroying the chart’s clean background with pervasive granular noise. CPIFuse (j) excessively smooths the dense lines, merging critical micro-scales and thereby defeating the resolution test’s purpose. In contrast, the proposed algorithm (k) achieved the optimal visual balance. By utilizing a multiscale denoising module based on local information entropy, it successfully filtered out polarization noise to present a clean background while maximizing the recovery and sharpening of dense high-frequency lines. Ultimately, the algorithm completely avoids overexposure, over-smoothing, and noise amplification artifacts, demonstrating superior detail protection and visual enhancement capabilities.

**Figure 11:**
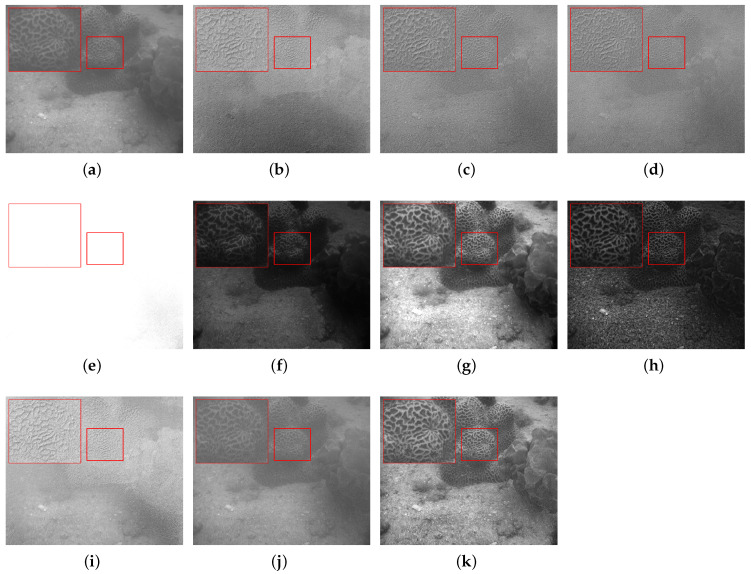
Scene 7: Underwater coral. (**a**) $S_{0}$. (**b**) DOLP. (**c**–**j**) Fusion images generated by methods 1 to 8, respectively. (**k**) Fused image from the proposed method. The red bounding boxes and the corresponding zoomed-in patches are added to highlight the detailed performance in texture recovery and noise suppression.

To further validate the algorithm’s performance in detail recovery and noise suppression within a complex real-world underwater scene, we selected a complex target scene from the U2PNet scene variation set for comparative analysis, as shown in Figure 11. Observing the source images, the intensity image ($S_{0}$) suffers from low overall contrast and distinct haziness due to underwater scattering, causing the complex reticular textures on the coral surface to appear blurry. Meanwhile, the degree of linear polarization (DOLP) image is severely contaminated by extreme background speckle noise, which almost completely submerges its physical structural information. Among the compared methods, Algorithms 1 and 2 fail to suppress the polarization noise effectively; their fusion results exhibit a strong grayish graininess and lack sharpness. Algorithm 3 suffers from severe distortion in brightness processing, resulting in massive overexposure (whitewashing) across the image that completely destroys all effective structural information. Algorithm 4 causes overall over-smoothing and a loss of high-frequency details during its denoising process, rendering the image excessively dark. Algorithm 5 exhibits obvious local overexposure, causing the textures in the brighter areas of the coral to blend together and lose depth. Algorithm 6, while increasing contrast, severely compresses shadow details and introduces unnatural structural artifacts around the object edges. The deep-learning baselines similarly fail in this complex environment. PAPIF (i) incorrectly amplifies the speckle noise, burying the coral under severe granular artifacts. CPIFuse (j) applies excessive smoothing, completely washing out the intricate reticular textures. In contrast, the proposed algorithm (k) achieves the optimal visual balance. Its information entropy-based multiscale denoising module not only successfully filters out the highly destructive polarization noise to maintain a smooth background, but also maximizes the recovery and sharpening of the fine textures on the coral surface without any overexposure, over-smoothing, or artifact interference. This comprehensively demonstrates its superior detail preservation and visual enhancement capabilities in real-world underwater environments.

Driven by the imperative to furnish a rigorous and objective appraisal of the algorithm’s proficiency in edge resolution recovery and low-polarization target restoration, this study strategically employs coral reef and resolution chart data from the public U2PNet and UPBD datasets for comparative analysis. The corresponding metric evaluations for the two images are presented in Table 10 and Table 11. It is worth noting that although some comparative algorithms (e.g., Algorithms 1, 2, 3, and 5) achieved abnormally high values in metrics such as Average Gradient (AG), Edge Intensity (EI), Spatial Frequency (SF), or Information Entropy (EN), this is actually because these methods failed to effectively filter out highly destructive speckle noise or introduced severe overexposure and halo artifacts during the fusion process. These meaningless noise particles and unnatural pixel fluctuations are mistakenly interpreted by gradient and frequency calculation formulas as “rich edge details,” resulting in artificially inflated objective metrics. This explains why these images exhibit extremely poor subjective visual quality despite performing exceptionally well in certain objective scores. In contrast, by strictly eliminating background noise and completely avoiding overexposure and artifact interference, the proposed algorithm achieves highly balanced and genuine objective metrics, truly realizing the perfect unification of high-quality physical structure recovery and human subjective visual perception.

Experimental results indicate that the proposed algorithm can effectively enhance the detail clarity and edge preservation capability of fused images across different scenarios, while well reflecting the overall structural characteristics. It achieves superior performance in both subjective visual effects and objective evaluation metrics.

### 3.3. Computational Complexity and Runtime Analysis

To comprehensively evaluate the algorithm’s potential for practical engineering deployment, we analyzed both its theoretical computational complexity and empirical execution time.

Theoretical Complexity: Unlike optimization-based physical models or deep neural networks that require heavy matrix multiplications and global iterative loops, the proposed framework relies strictly on feed-forward spatial operations. Let *N* represent the total number of pixels in the image (where N=W×H), and *K* represent the local window size for entropy calculation and filtering. The preprocessing (CLAHE) and the Information Entropy-Based Multiscale Denoising (IEB-MSD) operate locally, yielding a time complexity of $O(N\cdot K^{2})$. Since *K* is a small, fixed constant, the overall theoretical time complexity of the algorithm strictly simplifies to O(N). This linear complexity and the absence of global data dependencies allow the algorithm to process image data with low latency, making it exceptionally well-suited for subsequent practical deployment.

Empirical Runtime: To provide a standardized and reproducible comparison, we tested the execution time of the proposed method alongside the eight comparison algorithms. We randomly selected a subset of 30 images from the publicly available U2PNet dataset to compute the average execution time. All algorithms were executed on a PC equipped with an Intel Core i7 CPU and 32 GB RAM. The average execution times are summarized in Table 12. The average execution times are summarized in Table 12.

As demonstrated in Table 12, while maintaining superior fusion quality and structural fidelity, our method exhibits competitive computational efficiency. It significantly outperforms traditional complex multiscale transform methods (such as NSCT or Wavelet-based fusions in Methods 1 and 3) in terms of processing speed, achieving a highly favorable balance between enhancement performance and execution time.

### 3.4. Ablation Study and Parameter Analysis

To comprehensively evaluate the design choices within our proposed framework, this section presents a two-part ablation study. First, we perform a quantitative parameter analysis to determine the optimal values for the filtering scale and enhancement coefficients. Second, we conduct a module-level structural ablation to validate the indispensability of our core architectural innovations.

#### 3.4.1. Parameter Optimization Analysis

To rigorously justify the empirical parameters utilized in our proposed framework, we conducted a quantitative ablation study focusing on two critical variables: the standard deviation of the Gaussian low-pass filter (σ) and the detail enhancement coefficient (α). We utilized the representative “Underwater Agate Slice” scene (Scene 4) for this analysis, tracking five objective metrics: Information Entropy (EN), Standard Deviation (SD), Average Gradient (AG), Edge Intensity (EI), and Spatial Frequency (SF). The quantitative results are summarized in Table 13 and Table 14.

**Selection of Gaussian Filter Standard Deviation (σ):** As observed in the fixed-group (α=0.5), increasing σ from **1.0** to **2.0** brings significant structural gains, improving AG by **+3.14%**, EI by **+3.01%**, and SF by **+5.31%**. This indicates that a sufficiently large σ effectively isolates granular polarization speckle noise into the high-frequency band. However, as σ continues to increase to **2.5** and **3.0**, the improvement in structural metrics enters a stage of diminishing returns (with gains dropping to <1%). Concurrently, the Information Entropy (IE) decreases monotonically (from **7.4804** down to **7.4647**), and larger σ values inherently require larger Gaussian kernel sizes, directly increasing computational complexity. Consequently, σ=2.0 acts as the optimal inflection point, perfectly balancing structural enhancement, information fidelity, and computational overhead.

**Selection of Detail Enhancement Coefficient (α):** The coefficient α controls the intensity of injecting DOLP details into the low-frequency $S_{0}$ component. In the fixed-σ group (σ=2.0), setting α=0.2 yields the maximum values for AG, EI, and SF. However, it also produces the lowest EN and the highest SD, indicating an overly aggressive local fluctuation enhancement that indiscriminately amplifies fragmented textures and noise. On the other end of the spectrum, increasing α to **1.0** results in a noticeable drop in AG, EI, and SF, making the image visually softer and weaker in detail. The adopted setting of α=0.5 serves as a highly robust default middle ground. Structurally, it remains close to the optimal aggressive state (relative to α=0.2, the drops in AG and EI are <1%, and SF decreases by only **2.34%**), while keeping IE and SD more centralized and stable. This default moderate injection strength ensures excellent detail recovery without pushing the image into a noisy, over-sharpened state, ensuring stable performance across varying scenarios.

#### 3.4.2. Structural Ablation of Key Modules

To broaden the scope of our ablation study and comprehensively validate the necessity of the proposed architectural innovations, we conducted an additional module-level structural ablation analysis. We compared the full proposed framework against two structural variants using an additional underwater steel ruler scene (distinct from the scenes evaluated in previous sections). This specific scene, characterized by severe speckle noise and complex edge structures, was deliberately selected to maximally isolate and visualize the individual contributions of our noise suppression and artifact avoidance mechanisms: (1) **Removed IEB-MSD**: The novel Information Entropy-Based Multiscale Denoising module is replaced by bilateral filtering, disabling the entropy-guided noise discrimination. (2) **Removed Soft-Mask**: The low-frequency structure-guided soft mask ($M_{\mathrm{soft}}$) in the high-frequency fusion stage is removed, and a conventional blind “take-the-maximum-absolute-value” fusion rule is applied instead.

**Figure 12:**
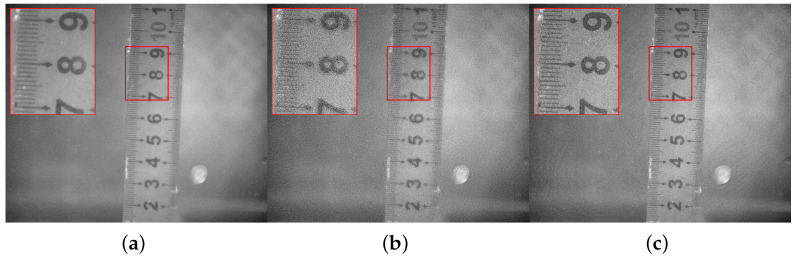
Underwater ruler. (**a**) Removed IEB-MSD. (**b**) Removed Soft-Mask. (**c**) Full algorithm. The red bounding boxes and the corresponding zoomed-in patches are added to highlight the detailed performance in texture recovery and noise suppression.

The quantitative metrics for these variants are summarized in Table 15, and the corresponding visual results are presented in Figure 12.

As demonstrated by the visual and quantitative results, removing the IEB-MSD module leads to a severe loss of high-frequency details. Traditional filters fail to distinguish between valid polarization textures and noise, resulting in a blurry fused image with the lowest Average Gradient (AG = 31.5024) and Spatial Frequency (SF = 36.6565).

Conversely, the “Removed Soft-Mask” variant completely exposes the limitations of traditional blind fusion. Although this variant yields abnormally high objective metrics (e.g., AG = 79.2538, EI = 349.4619), a visual inspection reveals that these inflated scores are entirely driven by the severe amplification of background granular noise and unnatural edge halo artifacts. In objective mathematical calculations, intense noise and rigid artifacts are often miscalculated as high gradients. Without the structural guidance of $M_{\mathrm{soft}}$, conflicting high-frequency components clash, severely degrading the visual naturalness.

The full proposed architecture successfully bridges this gap. It effectively suppresses artifact inflation while maintaining genuine structural sharpness, ensuring that robust noise suppression and precise, artifact-free detail restoration are achieved simultaneously.

## 4. Discussion

This paper proposes an information entropy guided and hierarchical adaptive fusion framework for underwater polarization image enhancement, aiming to address the issues of contrast degradation and noise interference caused by water scattering and absorption. The method first enhances the local contrast of the intensity image $S_{0}$ and innovatively applies a local information entropy based multiscale denoising to the degree of linear polarization DOLP image, in order to distinguish and preserve texture details associated with polarization characteristics. Subsequently, Gaussian filtering is used to decompose both images into low frequency and high frequency sub bands: for low frequency fusion, a detail injection strategy is adopted to extract structural information from the DOLP image to enhance overall contrast; for high frequency fusion, a “structure guided detail” fusion mechanism is introduced, which utilizes gradient information from the low frequency components to dynamically generate a soft mask, enabling phase aligned adaptive fusion and edge sharpening of multi source details. Finally, after sub band reconstruction and adaptive brightness adjustment, an enhanced image with natural appearance and clear details is produced.

Experimental results demonstrate that the proposed method significantly enhances image detail clarity and edge preservation capabilities on both self collected images and public datasets. It outperforms multiple mainstream fusion methods in objective metrics such as information entropy and average gradient, while maintaining favorable visual naturalness. Additionally, quantitative ablation studies validate the robustness of our optimized parameter configurations (σ=2.0, α=0.5), while theoretical and empirical runtime analyses confirm the algorithm’s strictly linear O(N) complexity and superior processing speed. Moreover, the Gaussian filter based decomposition and fusion mechanism adopted in this work ensures effective enhancement with relatively low computational complexity, offering a feasible solution for practical engineering applications. Future work could focus on further optimizing the fusion rules for low frequency sub bands and the Gaussian decomposition method to improve detail retention in semantic target regions.Furthermore, it is worth discussing the positioning of our framework relative to recent deep-learning (DL) approaches. While data-driven DL methods have demonstrated impressive visual restoration capabilities, this study deliberately focuses on a physics-aware, non-learning framework. The rationale is twofold. First, the application of DL in underwater polarization imaging is currently hindered by a severe scarcity of large-scale, high-quality paired datasets (i.e., degraded polarization images aligned with clear ground truths) required for robust network training. Second, data-driven networks are inherently susceptible to domain-shift vulnerabilities; they may generate unpredictable structural hallucinations or color artifacts when encountering highly turbid, unseen aquatic environments. For specialized tasks demanding strict physical and structural fidelity, such as underwater infrastructure inspection, our entropy-guided method ensures mathematically interpretable, artifact-free restoration. By strictly adhering to spatial polarization priors, our framework trades the unconstrained fitting power of DL models for absolute structural reliability and robust generalization across diverse, real-world underwater scenes.

## Figures and Tables

**Table 1:** Summary of core methodologies and fusion strategies for the compared algorithms.

Method	Core Methodology	Strategy
Method 1 [41]	DT-CWT/Curvelet/NSCT	Std. Max/Avg. (Transform)
Method 2 [42]	SWT, PCA	PCA (Transform)
Method 3 [11]	NSCT	Fuzzy Logic (Transform)
Method 4 [43]	Color Balance, Multiscale	Weight Map Fusion
Method 5 [14]	Multi-weight, Multi-granularity	Multi-weight Fusion
Method 6 [44]	UDCP, Variational	Model Restoration
Method 7 [45]	CNN, Attention Mechanism	Attention-Guided Fusion
Method 8 [46]	Transformer (Restormer), CNN	Cross-Attention Fusion
Proposed	Method in this paper	Method in this paper

**Table 2:** Determination strategies for key hyperparameters in the proposed framework.

Module	Parameter (Symbol)	Determination Strategy	Selection Rationale/Objective
Preprocessing	CLAHE clip limit	Determined via empirical grid search on diverse datasets.	To constrain local contrast enhancement, preventing the over-amplification of dark-region noise.
IEB-MSD	Gaussian standard deviation (σ)	Optimized via quantitative ablation study (detailed in Section 3.4).	To find the optimal cutoff frequency that isolates speckle noise while preserving low-frequency structural contours.
IEB-MSD	Local window size (*k*)	Analytically derived using the 3σ rule (k≈3σ).	To ensure complete coverage of the Gaussian function’s main energy, preventing spatial truncation artifacts.
Low-Freq Fusion	Background standard dev ($\sigma_{\mathrm{blur}}$)	Adaptively scaled based on the input image resolution.	A sufficiently large scale is required to completely filter out mid-to-high frequencies, leaving only a smooth illumination background.
Low-Freq Fusion	Detail enhancement coeff (α)	Selected as a robust default via structural metric ablation.	To achieve the optimal trade-off between maximizing detail saliency and strictly controlling global noise amplification.

**Table 3:** Objective evaluation metrics of different fusion methods for Scene 1 Underwater Steel Ruler. The best results are highlighted in bold.

Method	EN	SD	AG	EI	SF
Method 1	5.4369	10.5992	24.5029	108.2475	27.8984
Method 2	5.4305	10.5601	24.4250	107.9922	27.7691
Method 3	6.5568	23.5362	24.9374	109.9450	28.4854
Method 4	6.8194	29.6556	29.4664	128.2233	38.7545
Method 5	**7.5857**	**48.1983**	34.8686	146.7792	43.4143
Method 6	3.6014	3.3334	7.6116	33.1980	9.2569
Method 7	6.5841	24.0952	**54.8843**	**241.6337**	**62.6434**
Method 8	5.6502	12.2612	28.2911	130.0000	32.0865
Proposed	6.4061	30.9941	38.4372	167.8302	46.4215

**Table 4:** Objective evaluation metrics of different fusion methods for Scene 2 Underwater Stones. The best results are highlighted in bold.

Method	EN	SD	AG	EI	SF
Method 1	6.8289	47.2380	21.2216	92.1071	29.1722
Method 2	6.8456	47.1101	20.0986	87.2674	26.7593
Method 3	1.7680	48.0193	6.8046	28.8174	22.4478
Method 4	6.5741	34.7484	11.8683	49.8037	24.2207
Method 5	7.2224	47.3039	17.6307	71.8393	30.3419
Method 6	5.5315	41.0915	**25.6907**	**109.5551**	**39.1058**
Method 7	6.7641	**60.7053**	18.5280	79.4001	30.0916
Method 8	6.6152	44.2907	15.0539	64.9421	24.9484
Proposed	**7.2612**	46.3739	22.9326	97.7560	34.2697

**Table 5:** Objective evaluation metrics of different fusion methods for Scene 3 Underwater Plastic Bottle. The best results are highlighted in bold.

Method	EN	SD	AG	EI	SF
Method 1	6.8936	30.5448	**59.9849**	**263.1195**	75.2195
Method 2	6.8182	29.9340	52.8362	231.7241	71.6061
Method 3	5.5255	53.1427	46.3445	203.2185	69.4580
Method 4	7.1690	58.4166	13.9190	57.9214	27.7199
Method 5	**7.7688**	70.7753	17.4602	70.6351	30.3165
Method 6	6.3512	60.7066	31.7938	132.9077	54.1789
Method 7	7.7157	61.8096	57.7140	252.1795	**81.6782**
Method 8	7.6287	61.2268	35.8325	160.1943	44.8264
Proposed	7.5035	**75.5879**	53.7883	230.3042	70.2806

**Table 6:** Objective evaluation metrics of different fusion methods for Scene 4 Underwater Agate Slice. The best results are highlighted in bold.

Method	EN	SD	AG	EI	SF
Method 1	6.5137	23.3912	**31.8515**	**139.1738**	43.2791
Method 2	6.4835	23.1684	30.1891	131.9986	41.2564
Method 3	2.2870	29.7246	10.5987	46.7527	28.8866
Method 4	6.8726	34.2628	10.6052	45.0691	19.7341
Method 5	**7.7294**	**55.7116**	15.4249	63.1894	27.0152
Method 6	5.7845	36.6747	30.1190	127.7067	**58.3365**
Method 7	6.4727	21.8179	29.1663	128.2760	41.8971
Method 8	7.4222	44.3050	18.7789	83.7985	24.2255
Proposed	7.4708	53.7426	28.8373	123.4765	42.0688

**Table 7:** Objective evaluation metrics of different fusion methods for Scene 5 Underwater Algae Ball. The best results are highlighted in bold.

Method	EN	SD	AG	EI	SF
Method 1	5.9955	16.2219	23.8699	105.1514	30.5306
Method 2	5.9911	16.1700	23.7230	104.5834	30.2215
Method 3	7.0479	35.7536	23.8803	105.0584	30.5432
Method 4	6.3484	23.0605	12.5783	54.0928	19.4626
Method 5	**7.4061**	**44.6379**	17.5774	72.4958	26.5654
Method 6	3.2847	5.9979	8.6052	36.9354	14.6965
Method 7	6.5783	27.5872	**27.5113**	**120.6946**	**36.7839**
Method 8	5.7352	13.9938	15.3446	69.2949	18.8818
Proposed	6.4262	36.6023	27.3448	118.2039	35.6010

**Table 8:** Objective evaluation metrics of different fusion methods for U2PNet Dataset. The best results are highlighted in bold.

Method	EN	SD	AG	EI	SF
Method 1	6.5662	23.1290	**70.5971**	**308.1518**	**80.7891**
Method 2	6.5352	22.6301	69.7239	304.8117	79.7926
Method 3	0.8643	6.6621	6.8275	29.6927	19.8151
Method 4	6.8102	37.1100	23.8976	98.0737	35.3481
Method 5	**7.6000**	**54.0392**	37.9533	153.4807	50.9629
Method 6	5.9179	35.4857	39.7523	162.4765	54.8238
Method 7	6.8194	28.3544	66.5645	290.6699	78.4626
Method 8	6.7682	29.6271	39.2270	175.1403	45.0407
Proposed	7.2384	42.6746	54.9243	229.6980	66.9480

**Table 9:** Objective evaluation metrics of different fusion methods for UPBD Dataset. The best results are highlighted in bold.

Method	EN	SD	AG	EI	SF
Method 1	6.2144	23.8730	33.0774	135.7103	57.3548
Method 2	6.1569	22.8835	30.4022	124.6912	54.1803
Method 3	6.6679	55.8499	33.3461	136.6030	59.3058
Method 4	6.3363	46.3576	19.4312	79.0078	41.4732
Method 5	**7.2518**	**62.5009**	28.6671	116.3653	53.4415
Method 6	5.4846	36.6175	31.7417	128.9926	59.6022
Method 7	7.1281	50.4755	32.7002	133.8684	58.1281
Method 8	6.7212	41.3977	20.8680	85.7369	35.1312
Proposed	6.8175	59.8890	**40.2255**	**165.1007**	**66.5690**

**Table 10:** Objective evaluation metrics of different fusion methods for Scene 6 Resolution Chart. The best results are highlighted in bold.

Method	EN	SD	AG	EI	SF
Method 1	6.8376	38.2596	72.0231	294.0961	133.4719
Method 2	6.8390	36.7578	75.1864	306.7006	140.6597
Method 3	5.9468	88.3026	78.7040	321.2413	**166.0047**
Method 4	6.3714	63.1685	42.8390	172.6651	124.2031
Method 5	7.2320	**92.6969**	60.6868	244.8273	157.3547
Method 6	6.5683	57.9459	60.7636	245.2058	138.7108
Method 7	**7.4752**	72.4442	79.0754	**323.7942**	148.1070
Method 8	7.0205	66.2670	49.4185	201.8900	101.3250
Proposed	7.1024	90.5701	**79.2136**	323.4868	163.5684

**Table 11:** Objective evaluation metrics of different fusion methods for Scene 7 Coral Reef. The best results are highlighted in bold.

Method	EN	SD	AG	EI	SF
Method 1	6.5774	23.2000	**82.8465**	**361.5397**	**94.0711**
Method 2	6.5618	22.9626	82.4519	360.0743	93.6049
Method 3	0.5455	4.7981	4.8455	21.0565	18.2385
Method 4	6.5038	25.7451	23.0684	95.0016	34.9452
Method 5	**7.7164**	**57.0649**	42.3247	171.4822	54.0030
Method 6	5.6971	23.4152	42.1366	172.1796	54.9778
Method 7	6.6853	25.2105	77.5053	338.7686	90.6313
Method 8	6.6092	23.9569	43.7259	195.9634	49.6980
Proposed	7.1017	35.0754	58.7800	246.2515	70.1635

**Table 12:** The average execution times of Algorithms 1 to 7 on the U2PNet dataset.

Method	Average Runtime (in Seconds)
Method 1	664.0455
Method 2	3.5149
Method 3	611.1683
Method 4	22.6689
Method 5	1.6611
Method 6	9.3861
Method 7	2.0492
Method 8	6.5352
Proposed	1.5081

**Table 13:** Objective evaluation metrics for σ ablation (fixed α=0.5) on the single image pair.

σ	EN	SD	AG	EI	SF
1.0	7.4804	53.7355	27.9593	119.8695	39.9488
1.5	7.4753	53.7368	28.5879	122.5057	41.3747
2.0	7.4708	53.7426	28.8373	123.4765	42.0688
2.5	7.4676	53.7466	28.9063	123.6940	42.3896
3.0	7.4647	53.7591	28.8773	123.5096	42.5136

**Table 14:** Objective evaluation metrics for α ablation (fixed σ=2.0) on the single image pair.

α	EN	SD	AG	EI	SF
0.2	7.4649	53.7934	29.1191	124.5589	43.0753
0.4	7.4689	53.7611	28.9308	123.8371	42.4008
0.5	7.4708	53.7426	28.8373	123.4765	42.0688
0.8	7.4761	53.6791	28.5868	122.5001	41.1306
1.0	7.4789	53.6302	28.4352	121.9051	40.5471

**Table 15:** Objective evaluation metrics for module-level structural ablation.

Variant	EN	SD	AG	EI	SF
Removed IEB-MSD	6.4248	31.4961	31.5024	137.4958	36.6565
Removed Soft-Mask	6.6187	35.7805	79.2538	349.4619	93.0132
Full algorithm	6.5454	34.0249	61.6448	270.8380	72.9449

## Data Availability

The data presented in this study are available on request from the corresponding author.
